# Personalized strategies of neurostimulation: from static biomarkers to dynamic closed-loop assessment of neural function

**DOI:** 10.3389/fnins.2024.1363128

**Published:** 2024-03-07

**Authors:** Marta Carè, Michela Chiappalone, Vinícius Rosa Cota

**Affiliations:** ^1^IRCCS Ospedale Policlinico San Martino, Genova, Italy; ^2^Department of Informatics, Bioengineering, Robotics System Engineering (DIBRIS), University of Genova, Genova, Italy; ^3^Rehab Technologies Lab, Istituto Italiano di Tecnologia, Genova, Italy

**Keywords:** individualized, activity-dependent, *in-vivo*, humans, anatomical, functional, neurodynamics

## Abstract

Despite considerable advancement of first choice treatment (pharmacological, physical therapy, etc.) over many decades, neurological disorders still represent a major portion of the worldwide disease burden. Particularly concerning, the trend is that this scenario will worsen given an ever expanding and aging population. The many different methods of brain stimulation (electrical, magnetic, etc.) are, on the other hand, one of the most promising alternatives to mitigate the suffering of patients and families when conventional treatment fall short of delivering efficacious treatment. With applications in virtually all neurological conditions, neurostimulation has seen considerable success in providing relief of symptoms. On the other hand, a large variability of therapeutic outcomes has also been observed, particularly in the usage of non-invasive brain stimulation (NIBS) modalities. Borrowing inspiration and concepts from its pharmacological counterpart and empowered by unprecedented neurotechnological advancement, the neurostimulation field has seen in recent years a widespread of methods aimed at the personalization of its parameters, based on biomarkers of the individuals being treated. The rationale is that, by taking into account important factors influencing the outcome, personalized stimulation can yield a much-improved therapy. Here, we review the literature to delineate the state-of-the-art of personalized stimulation, while also considering the important aspects of the type of informing parameter (anatomy, function, hybrid), invasiveness, and level of development (pre-clinical experimentation versus clinical trials). Moreover, by reviewing relevant literature on closed loop neuroengineering solutions in general and on activity dependent stimulation method in particular, we put forward the idea that improved personalization may be achieved when the method is able to track in real time brain dynamics and adjust its stimulation parameters accordingly. We conclude that such approaches have great potential of promoting the recovery of lost functions and enhance the quality of life for patients.

## 1 Introduction

Neurological disorders are a growing global health concern, contributing significantly to the worldwide disease burden. While there has been a positive shift in communicable neurological disorders’ mortality and disability-adjusted life years (DALYs) from 1990 to 2019, the overall burden of neurological disorders is increasing due to the expanding and aging global population. Such a burden trends to vary significantly across geographical regions, influenced by genetic, socioeconomic, sociodemographic, environmental, and local healthcare factors, emphasizing the urgent need for enhanced global efforts, especially in economically disadvantaged regions (Ding et al., 2022).

In the quest to combat neurological disorders, neurostimulation plays a pivotal role. The ability to manipulate brain activity directly through external stimuli of various physical natures is a major driver in advancing innovative therapeutic strategies. In fact, neurostimulation techniques are widely used as neuroscientific investigation tools, in the development of neuroprostheses, and very successfully in the treatment of a broad variety of neurological conditions, such as Parkinson’s Disease (Cole et al., 2022), epilepsy (Krishna et al., 2016; Li and Cook, 2018; Dell et al., 2019; Davis and Gaitanis, 2020; Ryvlin et al., 2021), distinct cognitive (Bonizzato et al., 2023; Kolmos et al., 2023), and psychiatric dysfunctions (Nuttin et al., 2014), to promote functional recovery in brain injury patients (Bao et al., 2020), including stroke (Kolmos et al., 2023). On the other hand and despite its established status, the effectiveness of neurostimulation varies considerably among patients and across clinical trials of different applications. In fact, there is now mounting evidence that the patients’ individual particularities, even when minimal, may have a large impact on the effects of a given therapy, especially in the case of non-invasive methods (Non-invasive Brain Stimulation; NIBS) (Ovadia-Caro et al., 2019).

Along several decades of development, neurostimulation has benefited extensively from scientific progress and technological breakthroughs, such as a better understanding of the neurophysical basis of the interaction between electromagnetic fields and brain tissue (Nunez and Harth, 2005; Buzsáki and Vöröslakos, 2023), paradigm-shifting neuroscientific discoveries related to the processing of neural information (Varela et al., 2001; Buzsáki and Watson, 2012), innovative neural interfaces (Panuccio et al., 2018), and powerful signal processing methods, including the usage of artificial intelligence/machine learning tools (Fellous et al., 2019; Chandrabhatla et al., 2023), and neuromorphic strategies (Chiappalone et al., 2022; Christensen et al., 2022). By its turn, these allowed for the exploration of a series of novel stimulation paradigms, including temporally spatial complex stimulus patterns (Cota et al., 2023), and closed-loop modes of operation (Panuccio et al., 2016; Iturrate et al., 2018; Sellers et al., 2024). Collectively, these advancements are spurring a new era of disruptive neurostimulation, referred to as electroceuticals (Famm et al., 2013; Reardon, 2014, 2017), which can target specific nerves or neural pathways, addressing various chronic diseases and conditions, not limited to neuronal disorders. As a novel category of therapeutic agent, electroceuticals are at the very forefront of the broader field of neuroengineering. Devices like the NeuroPace RNS system (Razavi et al., 2020, neuropace.com), designed for epilepsy treatment through responsive neural stimulation, and the pioneering work of Galvani Bioelectronics (Guyot et al., 2019; Mullard, 2022, Science – Galvani Bioelectronics), focused on a stimulation platform for the blocking of neural signals via small bioelectronic implants, demonstrate the tangible outcomes of electroceutical therapy, which has rapidly progressed from a promising avenue of research to an important method of medical intervention.

In this review, we will revisit several studies on neurostimulation methods that, by adopting an electroceutical approach, can tune their protocols and parameters according to individual traits of the stimulation target and thus deliver more efficacious and safer therapy to patients. They are analyzed mainly under the perspective of the source of information for personalization (i.e., what patient parameter or biomarker defines the stimulation strategy). Considering the vastness of the bibliography on neurostimulation, this paper focuses on NIBS techniques. Yet, some specific invasive modalities and animal experimentation are also discussed due to their importance in supporting both the concepts of personalization and closed-loop strategies. Finally, here we are especially interested in reviewing personalized approaches for motor recovery in brain injured individuals, particularly invasive microstimulation techniques such as Activity Dependent Stimulation (i.e., ADS), which is based on a closed-loop approach to induce Hebbian-like plasticity (Jackson et al., 2006; Guggenmos et al., 2013). Such a view of the brain stimulation field yields, in our understanding, a convincing argument towards electroceuticals in general, and personalized strategies in particular.

## 2 Evidence in support of personalized interventions/treatments

The concept of personalization of neurostimulation as a means to address variability seen in its outcomes has recently gained a lot of prominence (Micera et al., 2020). Personalized stimulation aims to tailor protocols, parameters, and target selection to key characteristics of the patient, thereby optimizing treatment efficacy and improving outcomes, in an individualized medicine fashion (Kesselheim et al., 2023; Wessel et al., 2023). Historically, much of the original evidence in favor of personalization has come from distinct medical practices and seems to have been inspired by precision medicine spurred by genomics advancements during the ‘90s. At the beginning of 2000s, personalized medicine was a rapidly evolving healthcare field, as numerous benefits including improved patient outcomes and more efficient care were evident (Ginsburg and Willard, 2009). In 2013, a systematic review of the literature has led to the understanding that “personalized/individualized medicine” refer to a better “stratification and timing of healthcare” (Schleidgen et al., 2013). Such fine-tuning of treatment, or precision medicine (Ashley, 2016), is based on “biological information and biomarkers,” mostly at the molecular level, using technologies like genomics, proteomics, and metabolomics (Vogenberg et al., 2010; Goetz and Schork, 2018). Being a direct consequence of the spread of such ideas to non-pharmacological applications (PerMedCoalition, 2017), personalized neurostimulation is analogous to the general concept of individualized medicine in its fine-tuning of stimulus parameters. Differently from the former, though, its biomarkers stems largely from non-invasive imaging, electrophysiology, and/or other non-invasive imaging methods (system level), not those related to biochemical assessments (molecular level). Thus, in personalized brain stimulation, useful biomarkers can be of both neuroanatomical and functional nature, including position and size of neural substrates as revealed by imaging exams, or descriptive electrophysiological features.

Initial evidence in support of personalized brain stimulation stemmed from basic neurophysiological investigations of synaptic plasticity, the somatosensory and motor systems, perception/cognition, and neurodynamics in general. In 2006, Crochet and colleagues demonstrated that electrical stimulation at frequencies mimicking endogenous oscillation resulted in the effective induction of long term potentiation or depression in local neocortical networks of cats (Crochet et al., 2006). Using transcranial alternating current stimulation (i.e., tACS), Kanai and colleagues showed that visual phenomena such as phosphenes are evoked in a frequency dependent fashion, an effect putatively determined by the level of entrainment with oscillatory activity of the visual cortex (Kanai et al., 2008). For the motor function, other authors reported increases of corticospinal excitability, as assessed by the amplitude of magnetically-induced motor evoked potentials, only when tACS was applied to the motor cortex at physiological β-band frequency range (20 Hz), which happens to be a predominant oscillation observed in sensorimotor areas during quiescence in humans (Feurra et al., 2011). In a neurodynamical investigation, researchers used rhythmic bursts of transcranial magnetic stimulation (i.e., TMS), specifically tuned to preferential α-frequency, and electroencephalogram recordings (i.e., EEG) to demonstrate the entrainment of α-band oscillation in a cause-consequence relation (Coldea et al., 2022). Finally, some compelling results were provided by studies on neuromodulation of cognition using high-density tACS (i.e., HD-tACS). For instance, Helfrich and colleagues showed interhemispheric functional connectivity could be dynamically modulated according to the gamma-range synchronicity between neural oscillations and the bilateral application of HD-tACS. By its turn, this directly influenced human perception of shapes in an ambiguous motion task (Helfrich et al., 2014). In the same vein, Reihart and Nguyen demonstrated fast working-memory performance improvement in elders by applying HD-tACS with frequencies tuned in an individualized fashion (Reinhart and Nguyen, 2019). Nowadays, the importance of personalization is further emphasized in the clinical usage of personalized neurostimulation for epilepsy (Beumer et al., 2021), sensorimotor disorders (Gupta et al., 2023), cognitive function (Albizu et al., 2023), and dysfunction (Hunold et al., 2022; Reinhart, 2022; Aiello et al., 2023), and several other applications. In fact, researchers are actively exploring tailored approaches that leverage computational models, machine learning, and Bayesian optimization algorithms to optimize stimulation parameters and enhance treatment outcomes.

**Figure 1:**
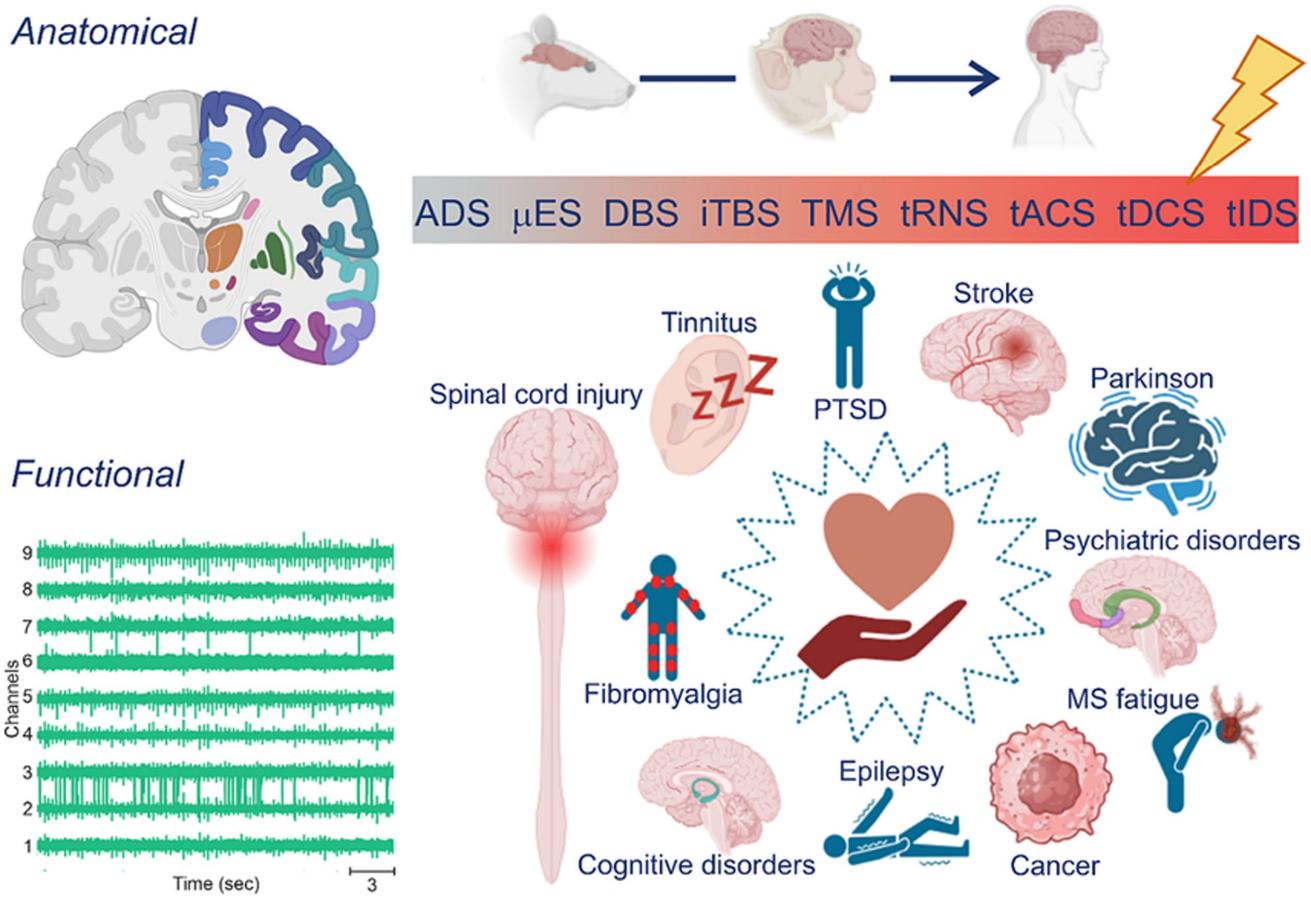
Personalized stimulation: from techniques to applications. (Left) Typical sources used to record target areas or patterns of interest are anatomical and/or functional. (Top, right) Electroceutical therapy typically involves the use of electrical stimulation to modulate neural activity in the nervous system. The application of electrical stimulation can be invasive (blueish hues), typically performed in animal models *in vivo* (e.g., rodents and primates) or non-invasive (reddish hues), most commonly done in humans. (Bottom, right) Neurostimulation can be used for various purposes, including pain management, treatment of neurological conditions, and rehabilitation.

In summary, these initial results suggested that by adopting personalization, which relies on individual characteristics (Figure 1), neural stimulation techniques could be more effective in promoting brain health and rewiring, inducing rehabilitation and enhancing the quality of life for individuals. In the next section, we will revisit major contributions in the literature towards the design of personalized neurostimulation approaches.

## 3 State-of-the-art in personalized neurostimulation

The field of personalized neurostimulation is now becoming a considerably broad area, almost as neuromodulation by itself is. As mentioned previously, it can be used as a therapeutical method for myriad neurological disorders including stroke, Parkinson’s disease, multiple sclerosis fatigue, epilepsy, and others (Figure 1, bottom right). Personalization can be achieved by choosing and adjusting electrode or coil geometry and/or position, the stimulation protocol, and the parameters of waveform or pulse morphology. They also differ on their level of development and technological/scientific maturity, which is partially correlated to its invasiveness. While some methods such as transcranial individual dynamics stimulation (tIDS) – a variation of the non-invasive tACS method – has been already tested on humans (Cottone et al., 2018), others such as activity dependent stimulation (ADS), which is dependent on the recording of multi-unit activity (MUA) and invasive micro stimulation (μES), has been carried out only experimentally in rodents (Figure 1, top right). In fact, variations of NIBS applied to humans currently represent most of the scientific/medical efforts in personalized stimulation. Thus, non-invasive methods are the focus of this review, while invasive alternatives (e.g., Deep Brain Stimulation (DBS) and intracortical microstimulation) will be discussed in the perspective of pioneering and explorative research that has been instrumental for the scientific and technological progress supporting non-invasive applications. Finally, personalization can be achieved by observing patient’s individual characteristics of two distinct natures, anatomical or functional, or even combinations of these aspects (Figure 1, left). This section focuses on this dimension, i.e., the nature of the informing parameter for personalized neurostimulation, while putting in due perspective the other important aspects previously mentioned.

### 3.1 Personalization based on anatomical information

**Figure 2:**
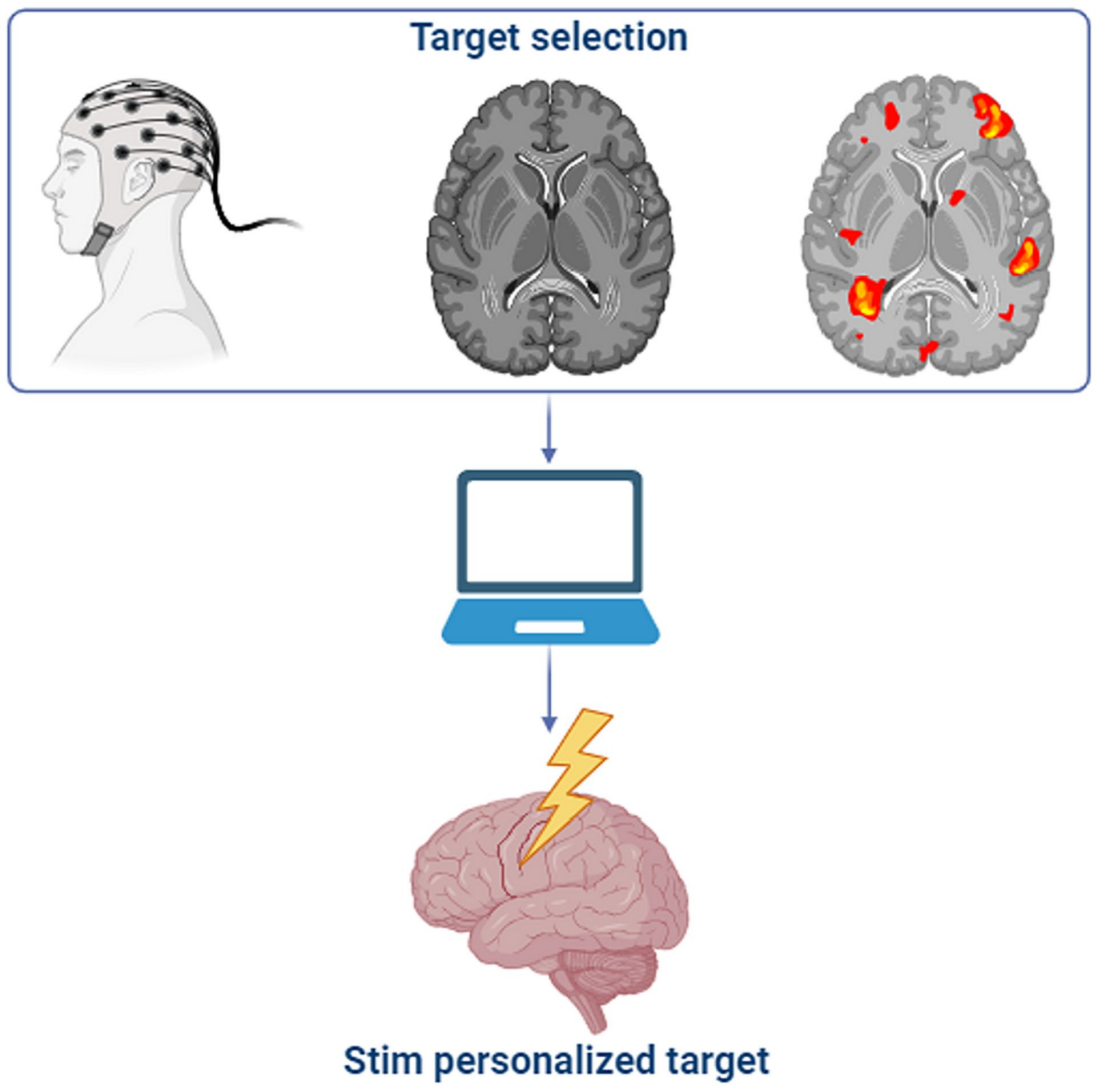
Obtaining individual neurodynamics. A general schematic flow of how to obtain/localize the source within the area of interest for each subject. (Top) Selection of target data (e.g., structural brain MRI, individual frequency, fMRI). (Middle) The personalized target selection is computationally elaborated (e.g., SofTaxic Neuronavigation, FSS, Hilbert transform, SMA activation analysis) in order to guide the procedure (e.g., stereotaxic, individualized frequency) for the personalization (e.g., electrode’s shape, localization/place). (Bottom) The personalized target is identified for stimulation.

A very plausible, even intuitive, strategy for the personalization of neurostimulation methods is to tune parameters according not only to a generic disorder-related brain substrate, but also mainly to neuroanatomical specificities of the patient being treated (Figure 2). In this approach, aspects considered are the precise mapping of the individual’s brain structures and their deviations from normality (pathologically or not), which directly influence the distribution of currents and electromagnetic fields generated by stimuli. The integration of information on both one’s anatomy and physical properties of stimulus source (e.g., coils or electrodes and their leads) may then be used in computational simulations in search of an optimal configuration of parameters. Fixed stimulation parameters across individuals, in a one-size-fits-all approach, are no longer sufficient as they fail to account for unique characteristics, thus leading to inconsistent outcomes.

A major effort in this direction has been the individualized modeling of current flow, which relies on volume conductor models to analyze the distribution of the electric field and electric current density within the complete head under stimulation (Hunold et al., 2022). This technique is based on the quasi-stationary approximation of Maxwell’s equations, assuming a linear connection between the electric field and current density, determined by the electrical conductivity of the tissues. Calculations can be performed on generic models of neural tissue, of the human head, or on real structural data obtained from the patient by non-invasive imaging methods. In this case, it takes into consideration important influencing factors such as skull defects, lesions, and age-related differences to better determine effective stimulation intensities and influential tissue compartments.

This rationale first appeared *circa* two decades ago and simultaneously in literature for both invasive and non-invasive techniques. In 2005, Butson and McIntyre used a Fourier finite element method for the calculation of electrical field distribution in cable models of myelinated axons to assess the effects of tissue and electrode capacitance in the application of DBS (Butson and McIntyre, 2005). Later, the same group also applied a finite element method for the reconstruction of the subthalamic nucleus of patients of Parkinson’s disease, with the goal to assess the volume of tissue activated (Butson et al., 2007). In a more recent study, Janson and Butson described an image-processing pipeline for incorporating Magnetic Resonance Imaging (MRI) and diffusion weighted imaging (DWI) content in a computational model of patient-specific DBS effects. The idea is to try different settings of electrode types, lead activation schemes, parameter selection, and precise positioning in a virtual and safe environment (Janson and Butson, 2018). This would maximize therapeutic effect while reducing risks associated with the neurosurgical procedure, collaborating with clinicians and researchers. Yet, a comprehensive review of such exciting developments on computational models of DBS and other invasive methods is beyond the scope of this review [for a detailed study see (Butson, 2012)]. Regarding non-invasive methods, one pioneering and influential work is the study of Miranda and collaborators who modelled current flow induced by non-invasive electrical stimulation by using finite element method (FEM) applied to a classic four concentric spheres model (Miranda et al., 2006). Their results showed that calculated current values and directions are very close to what can be found by *in vivo* and *in vitro* experiments, thus largely supporting usage of computational models for neuromodulation investigation. On 2012, Datta and colleagues were among the first to apply computational models of current flow in an individualized fashion by using true MRI data obtained from a stroke patient (Datta et al., 2011). They showed that lesion tissue has a considerable impact in shaping current flow, making a strong case for personalized strategies. Finally, in their recent review, Hunold and colleagues were able to find a series of studies in which current flow modelling was applied in the determination of transcranial electrical stimulation (tES) parameters applied to healthy individuals submitted to cognitive tasks, or patients with schizophrenia, major depression, Alzheimer’s, and Parkinson’s disease. Overall, findings of the reviewed studies pointed to the benefits of adopting a personalization strategy. Not only improved cognitive performance and amelioration of symptoms were observed, but the modelling allowed for the reduction of side-effects and better predictability of outcomes (Hunold et al., 2022).

In the particular case of transcranial direct current stimulation (tDCS), personalizing neuroanatomy seems to be a critical factor in optimizing outcomes (Hordacre et al., 2021). In fact, it seems to be imperative to consider individual differences, accounting for factors not only like cranial and brain anatomy, but also demographics, hormones, and genetics, when establishing standardized tDCS parameters using computational modeling techniques. For this, MRI neuroanatomical data can be also integrated in the modelling through methods like FEM, which has enabled predicting functional connectivity, motor-evoked potentials, and cortical blood oxygenation. In this context, machine learning techniques, such as a Support Vector Machine (SVM) model trained on a dataset of 14 healthy older adults have been employed to further optimize stimulation outcomes. Feature weights of the SVM were then utilized in a weighted Gaussian Mixture Model (GMM) to identify the most optimal electrode montage and applied current intensity, thus successfully converting tDCS non-responders to responders with optimized doses. This approach, as demonstrated by Albizu et al. (2023), holds the potential for personalized tDCS, promising enhanced efficacy and tailoring more effectively future interventions.

Computationally enabled personalization of stimulation parameters can also target electrode geometry and position. For instance, optimized electrode arrangements are necessary to account for brain lesion size and its impact on current distribution (Hordacre et al., 2021). A noteworthy example of such approach is the Regional Personalized Electrode (i.e., RePE), a specialized electrode designed to fit the unique cortical folding patterns of an individual’s brain (Tecchio et al., 2013; Cancelli et al., 2018b). RePE allows for precise shaping and positioning of tES electrodes, facilitating targeted stimulation of specific areas, such as the primary somatosensory (i.e., S1) and primary motor (i.e., M1) cortices (Tecchio et al., 2013). Authors behind this development report it enables electrode repositioning in multi-session protocols designed for home use, thereby improving treatment convenience and efficacy (Cancelli et al., 2015). Here, anatomical MRI is a suitable technique for personalized targeting of areas in the treatment of conditions like multiple sclerosis (i.e., MS) fatigue (Tecchio et al., 2016, 2022; Cancelli et al., 2018a). Personalized electrodes, such as RePE, have been found to result in higher electric field values, especially in the lateral regions, compared to non-personalized electrode (i.e., ReNPE) montages during tDCS (Parazzini et al., 2017). These disparities in electric field distributions significantly impacted a substantial portion of the cortical volume (Parazzini et al., 2017). Finally, Cancelli and colleagues demonstrated that applying tACS using RePE led to neuromodulation effects on both the hand and leg representations in the motor cortex (Cancelli et al., 2015). Conversely, such selective effects were not observed when ReNPE were used.

To address MS fatigue, neuroanatomical personalization has shown its significance also through interventions like Faremus (Cancelli et al., 2018b; Tecchio et al., 2022). Faremus employs personalized tDCS by fine tuning the shape of the anodal electrode based on MRI-derived individual cortical folding of the central sulcus to target the primary somatosensory cortex more specifically (i.e., S1), while leaving other areas unaffected. Previous trials have shown promising results on the efficacy of Faremus (Tecchio et al., 2016; Cancelli et al., 2018b). Recently, the authors reached an average improvement in fatigue levels of 27%, which is considered clinically significant (Bertoli et al., 2023). The ongoing adaptation and personalized nature of Faremus allow for long-term effectiveness and the potential to explore fatigue re-emergence over time. Moreover, personalized neurostimulation interventions have revealed alterations in neural connectivity as Faremus-induced neurostimulation have been demonstrated to have an impact in the connectivity between dominant and non-dominant corticospinal tracts. This emphasized the specific effect that personalized interventions can have on an individual’s neural connectivity and organization (Porcaro et al., 2019), highlighting the importance of tailoring treatments to target the central nervous system in conditions such as MS fatigue (Bertoli et al., 2023).

On the realm of magnetic stimulation, the combination of anatomical MRI and repetitive TMS (i.e., rTMS) has also demonstrated potential in enhancing cognition and addressing various conditions such as schizophrenia, obsessive-compulsive disorder (i.e., OCD), and alterations in the default mode network (i.e., DMN) in healthy individuals (Singh et al., 2019; Mantovani et al., 2021; Wu et al., 2021). In 2021, Mantovani et al. (2021), by using individualized rTMS sessions targeting the supplementary motor area (i.e., SMA), have found significant improvement in OCD symptoms, which persisted in time (e.g., for up to 3 months). Additionally, the study identified a decrease in connectivity between the SMA and subcortical brain regions associated with obsessions and compulsive behavior. Singh et al. (2019) explored personalized high-frequency repetitive transcranial magnetic stimulation (HF-rTMS) effects on the default mode network (DMN) in 23 healthy individuals. Their findings indicated reduced DMN-sgACC connectivity linked to lower harm avoidance and increased coupling between the right vStr and DMN associated with decreased self-reported negative mood. These results suggest that HF-rTMS might mitigate negative mood perception in healthy subjects, aligning with its observed effects in depression patients. Anatomical personalization becomes crucial when planning and performing stimulation to account for anatomical changes with spinal metallic implants in the context of spinal cord injuries (Guidetti et al., 2023). Inter-individual variabilities and variations in electrode placement must be carefully considered to ensure accurate stimulation and polarization of axon terminals. Personalized anatomy-based computational modeling can optimize the effects of transcutaneous spinal direct current stimulation (i.e., tsDCS) by considering the subject’s position during stimulation and understanding time-dependent effects and other influencing factors (Guidetti et al., 2023).

To address the variability issue in stroke patients specifically, Kolmos and colleagues conducted a randomized, double-blinded, sham-controlled trial investigating the efficacy of personalized tDCS in patients with subacute ischemic stroke and upper-extremity paresis (Kolmos et al., 2023). The trial involved 60 patients who received supervised rehabilitation training along with two sessions of 20 min of focal tDCS targeting the ipsilesional primary motor hand area (i.e., M1-HAND) per week for 4 weeks. In the personalized tDCS group, individual electrical field models were utilized to determine the optimal placement of the electrode grid on the scalp and the appropriate current strength at each cathode. This personalized approach ensured precise targeting of a physiologically relevant area with the right intensity. The control group received sham tDCS. The results demonstrated that the personalized tDCS group exhibited a significant improvement in the Fugl-Meyer Assessment of Upper Extremity (i.e., FMA-UE) score compared to the sham group at the end of the intervention. Additionally, by employing multimodal brain mapping techniques, the study provided insights into the mechanisms underlying the effects of personalized tDCS on the M1-HAND area.

In conclusion, the studies presented in this section provide clear evidence that optimizing and personalizing electrical stimulation techniques necessitate careful consideration of individual anatomical differences for each patient. Although many different approaches exist, modern neurostimulation should certainly be carried out with the fine tuning of its parameters (e.g., electrode position, electrode shape, etc.) as determined by neuroanatomical specificities. However, the importance of such information must be put in due perspective. While it is of uttermost importance to understand how brain structure and its variations – together with electrode configurations – may affect the propagation of electromagnetic fields and thus influence the activation of neural tissue, neurobiological function (and thus dysfunction) is by its turn largely determined by the dynamical interactions between brain areas. Personalization must, hence, extend beyond localizationism to encompass also physiological information as revealed by distinct measurements such as behavioral neural function assessment, fMRI, and electrophysiology (detailed in next section).

### 3.2 Personalization based on functional information

A first and evident strategy to deliver personalized stimulation based on information regarding function is to simply observe it as expressed by overall spontaneous or evoked behavior. Among others, Kesselheim et al. (2023) underscored the importance of functional personalization in optimizing non-invasive techniques, particularly TMS. They suggested characterizing individual motor cortex excitability and tailoring interventions based on factors like motor threshold, intracortical facilitation, and intracortical inhibition. This research underscored the intricate relationship between TMS parameters, cortical excitability, and diverse pathways in the corticomotor system. By monitoring changes in motor cortex excitability over time, the study emphasizes the importance of assessing treatment durability and developing strategies for sustaining personalized NIBS (Figure 2).

Besides behavior, electrophysiological recordings such as the scalp electroencephalogram (i.e., EEG) are also widely used for the personalization of electrical stimulation approaches. One such method has been demonstrated by Spooner and Wilson (2022), which is based on an individual’s peak gamma frequency of the EEG, derived from magnetoencephalography and a motor control paradigm in 25 healthy adults. These personalized peak gamma frequencies were then used for tailored sessions of tACS. Participants underwent four sessions of high-definition (HD)-tACS, including sham, low-, peak-, and high-gamma frequency stimulation over the primary motor cortex (M1) for 20 min while performing sequential movements of varying complexity. The study revealed that individualized tACS dosing over M1 significantly enhanced motor performance and learning, as evidenced by a notable reduction in the time required to complete motor sequences compared to nonspecific gamma-tACS in humans. Their study highlighted the significant impact of spectrally specific gamma range at low, peak, and high frequency tACS on motor execution measures compared to a sham condition. By exploring such individualized oscillatory signatures, their approach showed promising potential to enhance behavior and motor function recovery in clinical populations. In the same study, authors also stressed the importance of considering individualized dosing techniques and other factors beyond spectral specificity when optimizing NIBS.

Electroencephalography as a means for personalization has also been used in the tuning of tES parameters in the treatment of Parkinson’s disease (i.e., PD). Felice and colleagues conducted a randomized trial where they recorded EEG data and customized tACS frequency and electrode positions based on statistical comparisons with normative data (Felice et al., 2019). Participants received either tACS or random noise stimulation (i.e., RNS) for 2 weeks, followed by physical therapy. The study found that the tACS group showed a reduction in beta rhythm compared to the RNS group, along with improvements in symptoms like bradykinesia and cognitive function in PD patients. These positive changes were linked to a decrease in excessively fast EEG oscillations. In the same vein, Chung et al. (2019) investigated the effects of individualized intermittent theta burst stimulation (i.e., iTBS) on neurophysiological measures in the prefrontal cortex using TMS-EEG. Their study revealed that determining the individualized frequency for iTBS based on neuronal firing patterns could induce neurophysiological plasticity resembling long-term potentiation (i.e., LTP). These findings underscored the value of individualized iTBS and its potential for enhancing behavioral outcomes, further reinforcing the importance of personalization in brain stimulation techniques.

Given its impact on motor function and the underlying brain activity, observation and measurement of such phenomena would be instrumental also in the personalization of NIBS in stroke and brain injury applications. In fact, neuromodulation treatment of such patients may be one of the approaches showing the greatest variation in effectiveness, being influenced by factors such as age, gender, anatomy, stimulation parameters, symptom severity, lesion characteristics, stroke etiology, and time since symptom onset (Ovadia-Caro et al., 2019). Yet, such applications are considerably scarce in literature. For instance, Wessel et al. focused on the translation of NIBS into clinical practice for motor recovery after stroke. Their approach involved targeting the cortico-cerebellar system and employing a multifocal stimulation strategy, combining anodal tDCS applied to the primary motor cortex (i.e., M1) with hand-based motor training. Authors found no facilitation effects and highly variable stimulation responses. These findings, in their interpretation, are a major factor supporting the development of personalized strategies (Wessel et al., 2023). Other endeavors relied on the understanding that combining meaningful biomarkers of both anatomy and function can maximize the chances of successful motor recovery by personalized therapy in stroke rehabilitation (Ovadia-Caro et al., 2019). Building on this foundation, Beumer et al. (2021) developed a user-friendly software tool that streamlined research workflows and facilitated the analysis of personalized stimulation approaches. By integrating EEG source localization and tDCS optimization techniques, their tool maximized the overlap between the induced electric field and the intended stimulation target, particularly in cases of focal epilepsy.

Other authors have been employing personalization concepts in a broader non-disease-related perspective. In a particularly influential study, Cottone et al. (2018) developed a stimulation method called “transcranial individual neurodynamics stimulation” (i.e., tIDS), in which stimulation pattern is determined by the endogenous neurodynamics of the target region, as revealed by a technique called functional source separation (i.e., FSS) (Tecchio et al., 2007). In their 2018 study, FSS was applied to EEG recordings from healthy right-handed individuals performing isometric handgrip to identify neuronal subgroups of the motor cortex recruited during the task and to extract underlying neurodynamics. This activity was later mimicked in its waveform in an open loop stimulation procedure of the same activated areas (the so called tIDS), while corticospinal excitability was probed using TMS. The authors provided evidence that the excitability of the neuronal pool can in fact be influenced by such stimulation tailored to the endogenous activity of the target neuronal pool. They demonstrated tIDS was superior to other forms of standard tES in inducing cortical neuromodulation in individual participants. According to the authors, this novel approach has the potential to bring about a transformative change in the treatment of conditions like epilepsy, by specifically targeting the dysfunctional network associated with the disorder (Cottone et al., 2018).

Overall, the constant pursuit of reproducibility and optimization of tES has driven neurostimulation research towards definition of general guidelines and standards. In this sense, a recent study from Balderston et al. (2020) addressed challenges such as individual differences in brain structure and the absence of mechanistic rationales for stimulation parameters. To face this, they proposed a workflow, which incorporated fMRI-guided TMS stimulation, individualized volume conductor models, and optimized electrode placement, for paving the way to consistent and reproducible personalized tES interventions (Figure 2).

## 4 The importance of closing the loop

**Figure 3:**
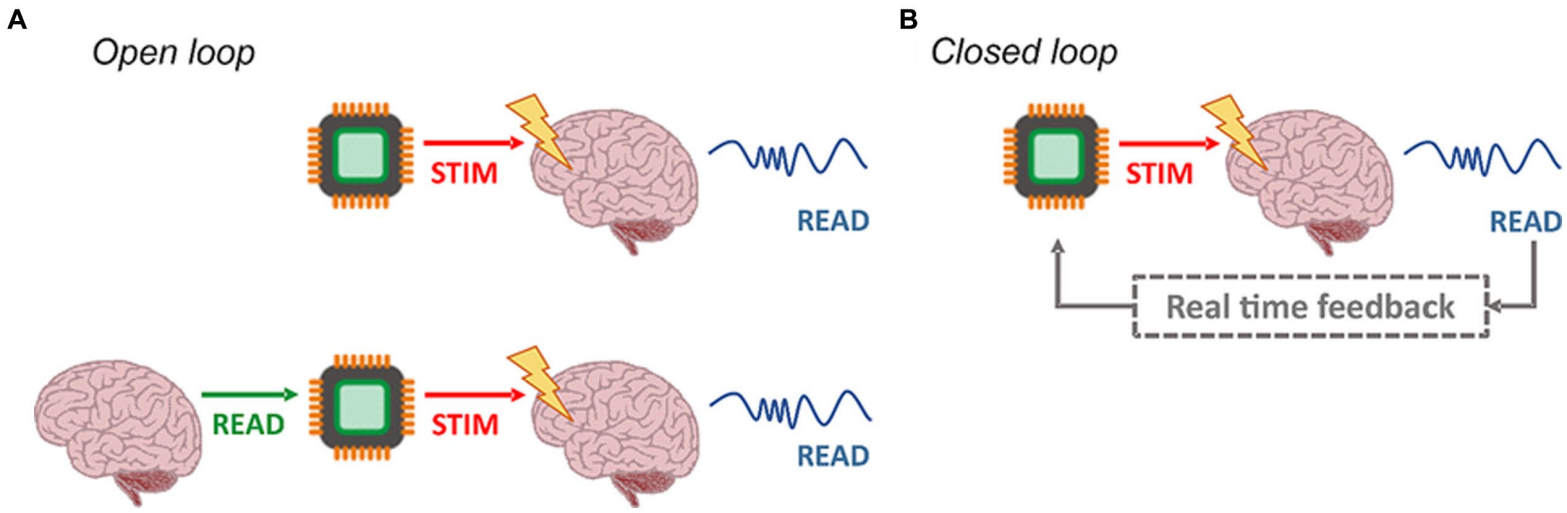
Open- and closed-loop architectures. **(A)** In the standard open-loop modality (top left), the delivered stimuli are not correlated to the network activity. It is possible to personalize the open-loop approach by designing a stimulation pattern, which reproduces the intrinsic dynamics of a target (bottom left). **(B)** On the contrary, closed-loop configurations rely on feedback. The signals from the network undergo processing, and specific features are extracted. Consequently, triggering events are generated, responsible for delivering stimulation pulses in accordance with the current network state.

So far, we have been discussing personalized neuromodulation that delivers stimulation which is fine-tuned to some relevant anatomical or functional aspects in a patient-specific manner. All the studies mentioned here, though, have also been carried out in an open-loop manner (Figure 3A). That means, while output stimuli settings are adjusted based upon the input features extracted from the neurobiological assessment of the patient, such input–output relationship is static and fixed in a time point in the past (Figure 3A). Such approaches are, thus, unable to react to important changes in the underlying neurodynamics that the patient may undergo. The incorporation of real time closed-loop techniques, in which stimulus can be adjusted based not only in past measurements but also according to brain state changes as they happen, represented an important breakthrough to neuroengineering systems in general (Figure 3B) (Guggenmos et al., 2013; Vato et al., 2014; Chiappalone et al., 2022). Enabled by nowadays unexpensive hardware systems for digital signal processing – e.g. microcontrollers, DSP microprocessors, application specific integrated-circuits, and FPGAs – it substantially improved systems’ performance – therapeutic efficacy and energetic efficiency – and safety (Zanos, 2019). This is because, in closed-loop systems, stimulation is delivered in an optimized way, only when and where it is really required, minimizing energy transfer from power sources to the neural tissues which, consequently, decreases risks. Finally, such mode of operation also spurred new and different forms of brain-technology interactions, such as neuromorphic and biohybrid prosthesis (Chiappalone et al., 2022).

In any case, the number of studies on the usage of closed loop neuroengineering systems for neuroscientific investigation and/or as means of neurological therapy, both in animal experimentation and pre-clinical/clinical trials, have been steadily increasing over the years. It has been now demonstrated that, in fact, application of electrical stimulation which is directly determined, in real time, by recorded electrophysiological activity can: promote both plasticity and motor recovery in the cases of stroke or traumatic brain injury (i.e., TBI) (Cheng et al., 2014); induce significant changes in walking performances in cerebral palsy (Zahradka et al., 2021); block pathological activity and promote the physiological one within the damaged tissue on Parkinson’s disease (Rosin et al., 2011) or epilepsy (Berényi et al., 2012; Skarpaas et al., 2019; Cota et al., 2021), and promote motor recovery after spinal cord injury (Ganzer et al., 2018). For the treatment of Parkinson’s disease, researchers usually monitor the power of the beta band to trigger DBS, such as the work of Little and colleagues (Little and Brown, 2012; Little et al., 2013). In the same vein, DBS triggered in real time by aberrant increases of ERD in the motor cortex has shown robust therapeutic effect in humans with tremor (Herron et al., 2015). Finally, preliminary results from Ganzer and colleagues suggest real time adaptive closed loop stimulation of the vagus nerve (i.e., CLV) as a promising strategy in clinical treatments for a range of neurological pathologies such as stroke, peripheral nerve injury, tinnitus and post-traumatic stress disorder (Ganzer et al., 2018).

The science and technology of closed-loop systems have seen particularly notable progress with animal experimentation, with both non-human primates and rodents. In 2006, Jackson and collaborators reported that neural activity can be intentionally modified, and visual feedback can assist in this modulation, creating a pseudo-closed-loop brain-machine interface (BMI) (Jackson et al., 2006). Recently, there has been a growing trend to integrate closed-loop systems into the design of BMI (O’Doherty et al., 2009, 2011; Venkatraman et al., 2009), which record neural activity, decode it, and provide stimulation to a peripheral target. Such systems can bypass neural damage at the spinal cord level (Moritz et al., 2008; Pohlmeyer et al., 2009; Ethier et al., 2012; Bouton et al., 2016). In addition, researchers have created devices that enable communication within the cortex in healthy subjects, including activity-dependent pairing to alter the motor output of neurons in M1 (Jackson et al., 2006). Dzirasa et al. (2011) and O’Doherty et al. (2011) have demonstrated that non-human primates can use M1 activity to drive motion towards a somatosensory cortex stimulation trigger. These examples employ brain-machine-to-brain interfaces (i.e., BMBIs) for communication within a cortical area or between areas in healthy subjects, which may be affected by small changes in synaptic efficacy between the recorded and stimulated sites.

Moving forward to incorporate personalization in closed loop neurotechnologies, applications in epilepsy plays a particularly relevant role. This is due to several reasons. First, seizure suppression neurostimulation methods are very diverse and rely on both invasive (Velasco et al., 2022; Xue et al., 2022) and non-invasive (Berényi et al., 2012) methodologies, the former representing a much better developed and wider frontier of investigation. Second, both modalities have closed loop solutions, including an FDA-approved system: Neuropace RNS^™^; the importance of which will be further detailed in the next section. Third, neurotechnology is largely used in epileptology as a means not only to deliver seizure suppression stimulation, but also to detect and even predict seizures (indeally in closed loop fashion). On top of all this, some very influential groups and literature have already established that inter-individual variability is a major issue regarding both outcomes of antiepileptic neurostimulation (Li and Cook, 2018) and electrographic biomarkers for seizure anticipation (Freestone et al., 2017). Naturally, this understanding led to several proposals of personalization of such technologies. Sisterson and colleagues have put forward the idea – and also implemented database and processing pipeline – of using data and evet logging capabilities of the RNS system for the automatic selection of a stimulation protocol among a diversity of options (Sisterson et al., 2019). More specifically, protocols would differ in terms of which specific lead contact would deliver the stimulus according to the spatial and temporal distribution (contact-wise) of aberrant epileptiform activity observed during detection. The rationale for this approach is based on the concept of choke points – or key propagation points – in neural circuits responsible for the generation of epileptic phenomena. Targeting such points (neural substrates) would be an optimal strategy for suppressing seizures (Piper et al., 2022). On the other front, patients used a mobile diary app to log self-reported seizures and thus create a forecasting profile based on low and high-risk state (Karoly et al., 2020). Authors reported that, in fact, seizures occurred mostly during high-risk periods. On a following study, the same group compared the predictive power of non-individualized (temporal features and weather) with individualized data (sleep assessment) using a Bayesian approach (Payne et al., 2021). They discovered that although incorporating individual characteristics is helpful for predicting the likelihood of seizures, it wasn’t better than using all available data. Finally, Viana and colleagues used intra-subject deep learning classifiers to assess ultra-long-term semi-invasive (subcutaneous) electrographic recordings to perform pseudoprospectively seizure forecasting (Viana et al., 2022). They found that sensitivity of the method ranged from 64 to 80% and that the outputs closely followed patient-specific circadian patterns of seizure occurrence.

These studies indicate that closed-loop technology can add a whole new level of individually tailored electroceutical therapy. Yet, the extent to which these real-time systems are being used as means to provide true personalized neurostimulation must be put into perspective. For a patient-tailored approach, the loop action must go beyond simple detection/trigger relationships, as carried out by virtually all the previously mentioned studies. As per definition, it is necessary to incorporate into the system direct measurement of the brain state or function as a main driver of the feedback action of the loop. Alongside with its effect on the external (in reference to the brain) world, including on the organism itself, such configuration of the method has been described in past work as “behavior-in-the-loop setups” (Zrenner et al., 2016). To these authors, usage of such configuration allows NIBS (and we add, other neurostimulation strategies) to be “coupled to endogenous brain activity in functionally defined brain networks in real time,” being an improved way to truly personalize therapy. In this perspective, it may be hard to properly assess the level of contribution for personalized neurostimulation provided in the literature of closed-loop systems, both from human and animal experimentation. We tend to agree with the notion that solely connecting the raw electrophysiological signal of the brain (i.e., the output) to the neurostimulation inputs in a simplistic threshold detection/trigger stimulation fashion, does not fully configure personalized therapy. On the contrary, usage of truly descriptive information of brain state and function to adjust stimulus parameters in real time is mandatory.

On the following section, we describe Activity Dependent Stimulation (ADS), a form of closed loop neuroengineering approach aimed at inducing Hebbian-based plasticity to promote brain rewiring and motor rehabilitation in animal models *in-vivo*. Being dependent and performed on the unitary level of neuronal activity (i.e., of the single neuron), ADS may represent a true approach towards personalized stimulation once it uses a powerful biomarker of brain dynamics: the temporal structure of its neuronal firing.

### 4.1 Activity dependent stimulation as one personalized closed loop neurostimulation approach

**Figure 4:**
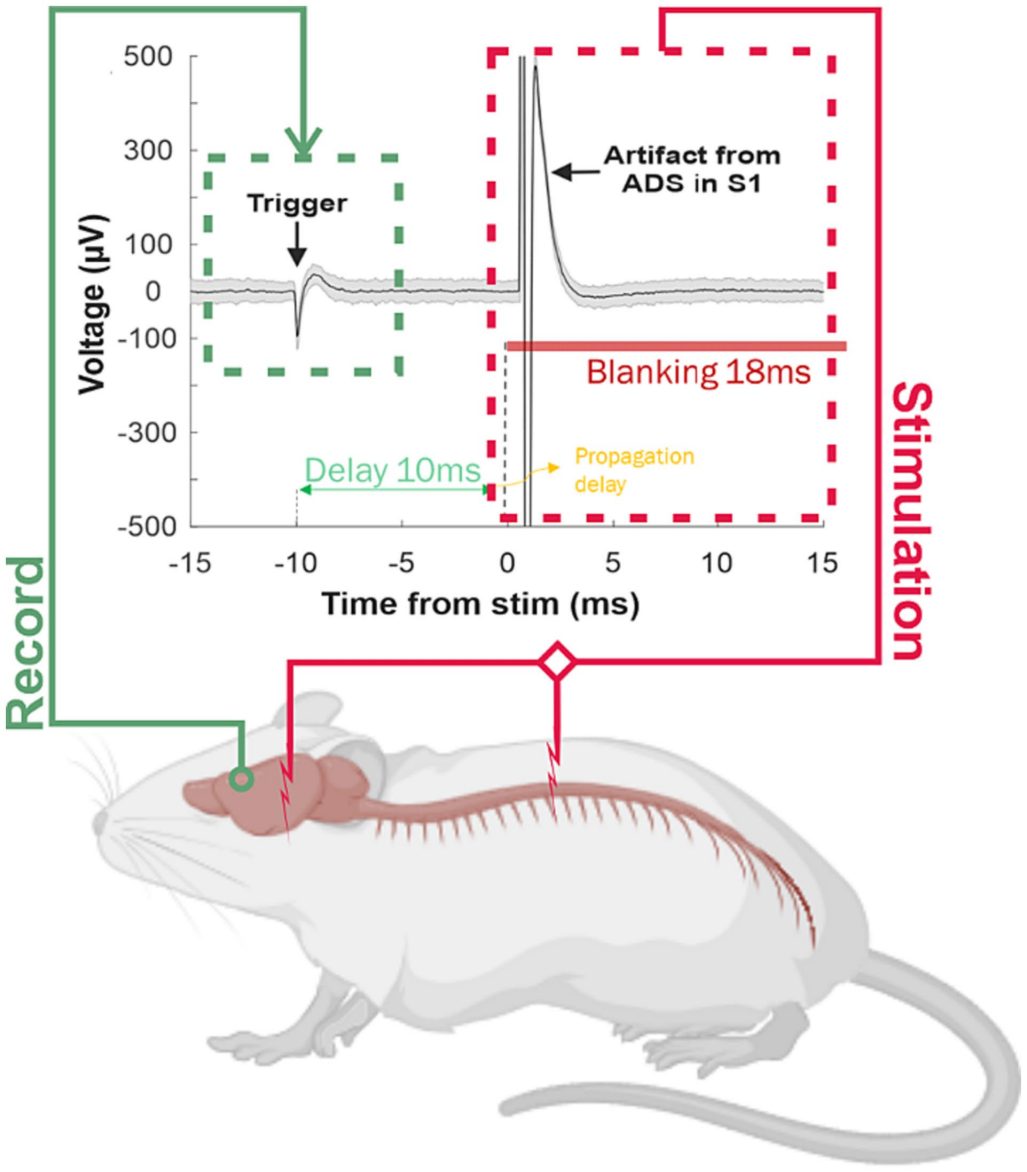
Overview of the ADS stimulation paradigm. A stimulation trial, during which a single-unit activity was detected on a single channel within the premotor cortex. The latency between the spike detection in the trigger area and delivery of a stimulus pulse was set at 10 ms (2.5 ms spike processing time, 7.5 ms imposed delay). To prohibit stimulus-activated spikes and stimulus artifacts from triggering stimulation, a short blanking period (28 ms) followed each stimulus. This activity was used to trigger a single stimulation pulse in the stimulation area [i.e., S1 (Guggenmos et al., 2013) or the ventral horn of the thoracic spinal cord below the level of the injury (Borrell et al., 2020)] in order to induce changes in the strength of the resultant activity through Hebbian mechanisms. A stimulus was triggered each time a user-selected neuronal spike profile was recorded from a single recording site in the motor cortex.

ADS was originally proposed by Guggenmos et al. (2013) as a first attempt to use a BMBI for the treatment of traumatic brain injury (i.e., TBI). The idea was to establish an alternative cortico-cortical communication between distant areas within the sensorimotor cortical loop using a neuronal-guided stimulation. ADS uses the occurrence of action potentials in one neuron to trigger stimulation at another location or electrode site at a fixed time delay, relying on the concept of Hebbian plasticity, in which repeated concomitant firing of two neurons will strengthen the connection between them (Figure 4). The authors demonstrated better efficacy in terms of behavioral recovery by comparing the performance of ADS with that of a randomized version of the protocol (random stimulation, RS) using a simple reaching pellet task in rats. The interpretation was that, by artificially pairing spike-firing in one population of neurons with focal electrical stimulation of a second population of neurons, it may be possible to re-shape the efficacy of specific neural pathways *in vivo* (Jackson et al., 2006; Rebesco et al., 2010; Rebesco and Miller, 2011; Guggenmos et al., 2013; Nishimura et al., 2013).

While the behavioral outcomes of microstimulation in sensory and motor regions has been characterized, few studies had examined the long-term effects of repetitive microstimulation on neuronal activity in the broader network of interconnected brain regions. Averna et al. (2020) investigated the effects of open-loop (i.e., Random stimulation, RS) and closed-loop (i.e., ADS) intracortical microstimulation on neural activity in distant cortical areas on healthy anesthetized rats. Overall, the study suggests that ADS has the potential to modulate neural activity in distant cortical areas in a direction-selective manner and could be a promising approach for the treatment of movement disorders. Moreover, in Averna et al. (2021), authors aimed to determine whether neurophysiological changes can be induced through cortical stimulation in healthy, ambulatory animals. The results confirmed the previous study (Averna et al., 2020) showing ADS was more effective than RS in entraining the network response by evoking stimulus-associated spiking activity, and only ADS induced increased synaptophysin expression within the region of stimulation.

To better understand the mechanistic properties of ADS for modulating activity, other studies investigated its effect on neuronal firing patterns in rats with focal ischemic lesion in the motor cortex (i.e., M1 or CFA). Their findings suggest that ADS can rapidly alter intrinsic neural activity after an injury, which could be effective for modulating activity in the acute periods after injury (Averna et al., 2022; Carè et al., 2022). Additional evidence supporting the use of ADS as a viable therapeutic method also after a spinal cord injury (i.e., SCI) were shown in Borrell et al. (2020), after demonstration that it could enhance synaptic efficacy in remaining pathways between the motor cortex and spinal cord. It was also shown that ADS led to a rise in cortically evoked spikes in spinal cord neurons, particularly at certain combinations of spike-intraspinal microstimulation delays and pulse numbers. Furthermore, the effectiveness of descending motor pathways increased across all dorsoventral depths of the hindlimb spinal cord.

In a recent study, Hudson et al. (2023) investigated the effectiveness of using ADS to induce rapid and long-lasting motor recovery in rats with traumatic brain injury. The researchers found that ADS improved motor performance during the sub-acute phase after injury, with significantly better reaching success compared to control animals after 1 week of stimulation and approaching pre-injury levels after 2 weeks. However, the study also showed that there was a significant decline in the speed and level of recovery in animals treated 3 weeks after injury, indicating the importance of early intervention. The researchers suggested that further studies are necessary to understand whether the recovery using ADS can extend beyond the sub-acute stage into the chronic period. Interestingly, the study found that stopping ADS after 4 weeks of treatment did not negatively impact the performance on the reaching task the following week. Researchers hypothesized that this may be due to the intrinsic neural pathways that were activated by ADS, which led to long-lasting structural and functional changes that superseded the dependence on the therapy. Furthermore, the study proposed that using intrinsic neural activity to drive stimulation rather than a forced, externally generated pattern could activate innate mechanisms of neuroplasticity and result in a more significant response. The researchers suggested that ADS could be an effective solution for promoting motor recovery during the sub-acute phase after injury, as this is the period when neuroplasticity is most active. In contrast, the chronic injury phase is associated with a reduction in the expression of neuroplastic mechanisms and a decreased ability for spontaneous or therapy-driven recovery to occur.

These findings could have important implications for the development of personalized closed-loop strategies for the treatment of movement disorders. By selectively modulating the activity of neurons that are relevant for movement, they could lead to more effective and targeted treatments. In any case, functionally informed closed-loop stimulation such as ADS currently represents a powerful platform to devise novel ways of personalized neurostimulation. Naturally, further research is needed to determine the specific effects of the method on behavior and to optimize its parameters for clinical use.

## 5 Discussion

The intuition that customizing medical treatment not only to address the precisely diagnosed issue but also to account for the individual characteristics of the patient can lead to improved effects, while also preventing undesired ones, has long been recognized. Unfortunately, there were no proper tools to fully account for the variability stemming from the multiple possible combinations among all the influencing factors related to physiological and pathological mechanisms. This is particularly true and aggravated in the case of neurological disorders, given the complexity of the nervous system. If there is one consensus to be reached in this scenario is that the idea of “one size fits all” should be taken with a grain of salt or maybe even abandoned altogether.

Fortunately, on the other hand, recent years have seen sound scientific and technological progress that may be able to cope with this reality. New and powerful diagnostic and investigational tools, such as genomics assessments and noninvasive imaging – and so many others – have enabled an unprecedent level of detailed scrutiny of the patients’ conditions and particularities. Advanced medical knowledge has allowed precise interpretation of the new data. By its turn, this new level of understanding is spurring myriad novel strategies for delivering therapy. It started *circa* a couple of decades ago with pharmacological treatment with new generation drugs that can target neurochemical substrates with greater specificity for improved effects. More recently, the approach towards personalization started spreading to non-pharmacological methods, one of them being neurostimulation.

In this review, we revisited a series of attempts towards such personalized neurostimulation with the main goal of drawing a panorama of the state-of-the-art in the field. By assessing literature on both clinical and pre-clinical studies, we were also able to get a better grasp on how such noble efforts adhere and embrace the state-of-the-art in neurotechnology in general (for a review see Chiappalone et al., 2022). Here, an important focus was given to closed-loop solutions. From the reviewed literature, it becomes clear that a significant portion of the efforts towards personalized stimulation is being carried out with the many different methods of NIBS at the clinical trial level. Personalization was obtained by specifically tuning electrode format, stimulation target, and stimulation parameters such as frequency of pulses and even wave morphology. These choices were mostly derived from static past assessment of the individuals’ particular traits as assessed by pre-treatment behavioral, imagining, and electrophysiological examinations. The studies reported varying degrees of success but at the same time an optimistic note supporting the choice for personalization was very common. Whether the approach will resist informed skepticism and additional investigations in the future years is yet to be determined.

On the other hand, the option for closed-loop system, which is arguably preferable, was virtually absent in the clinical studies. Although the reasons for this are unclear, it is plausible to suppose this is due to its methodological complexities, which also raise ethical concerns. Therefore, it is natural that such attempts are currently being carried out mostly in animal experimentation, at the preclinical level. Likely for similar reasons, studies involving animals provide opportunities for invasive recording and stimulation methods. These methods are less affected by the numerous neurophysical factors that can influence both the interpretability of recorded signals and the ability to target specific anatomical areas and functional processes. The path to follow is thus evident and involves further investigations to improve closed-loop techniques of personalized neurostimulation in all the relevant dimensions of efficacy, efficiency, and safety. This and the attempt to translate findings and methods from animal research to non-invasive methods will certainly favor the successful translation to the clinical setting.

Finally, here we reviewed the growing literature on ADS to make the case that it is one such promising avenue. In our understanding, the experimental method is: (1) a truly real time closed-loop system that; (2) can track closely the ever-changing neurodynamics which; (3) is directly related by its nature to very descriptive biomarkers of brain states and its specificities. The principle of a one-to-one relationship between neuronal spike and stimulus pulse not only is an efficient strategy to capture in detail the specific activity pattern of the brain under experimentation, but also to deliver the very same dynamic back to the system. Moreover, the fact that there is a fixed delay in the recording and stimulation loop tuned to a plasticity-inducing time window, in a Hebbian-like fashion, brings great potential for brain rewiring therapy. This is certainly useful in scenarios such as promoting robust motor rehabilitation after stroke, TBI or in the treatment of other disconnection disorders, as evidence now strongly suggests. Naturally, there is still much work to do before making the bridge to clinical trials, as ADS has met some limitations, particularly the lack of persistence of the beneficial effects after the interruption of the therapy. We propose that this may be improved by incorporating other descriptive biomarkers, including neuroanatomy (particularly of the lesion) and additional electrophysiological features spanning different temporal and spatial scales, such as global forebrain neurodynamics and the sleep–wake cycle architecture. Moreover, further investigations of the true detailed mechanisms of ADS are mandatory. Indeed, this perspective naturally connects clinical trials of personalized NIBS with experiments involving closed loop microstimulation in animals, particularly ADS. This connection suggests a potential paradigm shift from static biomarkers to dynamic closed-loop evaluation of neural function in personalized neurostimulation.

In conclusion, we can say that, given the plethora of pathologies affecting the nervous system and the significant variability with which they manifest, the personalization of the therapeutic approach becomes essential to maximize benefits and restore brain functions. There are many virtuous examples of electroceutical therapies in the literature, some still in their infancy. Investing in studies and research aimed at translating such an approach from preclinical models to humans is necessary to promote the recovery of lost functions due to brain damage and, ultimately, enhance the quality of life for patients.

## Author contributions

MCa: Writing – review & editing, Writing – original draft, Visualization, Conceptualization. MCh: Writing – review & editing, Writing – original draft, Supervision, Funding acquisition, Conceptualization. VC: Writing – review & editing, Writing – original draft, Supervision, Funding acquisition, Conceptualization.
